# Circ_0007841 promotes the progression of multiple myeloma through targeting miR-338-3p/BRD4 signaling cascade

**DOI:** 10.1186/s12935-020-01475-6

**Published:** 2020-08-08

**Authors:** Yan Wang, Quande Lin, Chunge Song, Ruojin Ma, Xiaojie Li

**Affiliations:** 1 Department of Hematology, The Fifth Affiliated Hospital of Zhengzhou University, No.3 Kangfuqian Street, Zhengzhou, 450052 Henan China; 2 Department of Hematology, Henan Cancer Hospital, Zhengzhou, 450052 Henan China

**Keywords:** Multiple myeloma, circ_0007841, miR-338-3p, BRD4, Exosome

## Abstract

**Background:**

The pathogenesis of multiple myeloma (MM) is not completely known. Uncovering the potential mechanism of MM initiation and progression is essential for identifying novel diagnostic and therapeutic targets. Herein, we explored the function and the working mechanism of circular RNA circ_0007841 in MM progression.

**Methods:**

Quantitative real-time polymerase chain reaction (qRT-PCR) was employed to detect the expression of circ_0007841, microRNA-338-3p (miR-338-3p) and bromodomain containing 4 (BRD4). Cell proliferation ability was analyzed through cell counting kit-8 (CCK8) assay, colony formation assay and flow cytometry. Transwell assays were conducted to measure the migration and invasion abilities of MM cells. Cell apoptosis was also assessed by flow cytometry. The interaction between miR-338-3p and circ_0007841 or BRD4 was confirmed by dual-luciferase reporter assay and RNA-pull down assay.

**Results:**

Circ_0007841 was highly expressed in bone marrow (BM)-derived plasma cells of MM patients and MM cell lines than that in healthy volunteers and normal plasma cell line nPCs. Circ_0007841 promoted the proliferation, cell cycle and metastasis and impeded the apoptosis of MM cells. miR-338-3p was a direct target of circ_0007841 in MM cells and circ_0007841 accelerated the progression of MM through targeting miR-338-3p. BRD4 could directly bind to miR-338-3p in MM cells and miR-338-3p exerted an anti-tumor role through targeting BRD4. Circ_0007841 promoted the activation of PI3K/AKT signaling via miR-338-3p/BRD4 axis. Exosomes generated from mesenchymal stromal cells (MSCs) elevated the malignant behaviors of MM cells via circ_0007841.

**Conclusion:**

Circ_0007841 acted as an oncogene to promote the proliferation, cell cycle and motility and restrain the apoptosis of MM cells through sequestering miR-338-3p to up-regulate the expression of BRD4.

## Background

Multiple myeloma (MM) is a kind of hematologic cancer featured by malignant proliferation of plasma cells [1]. The therapeutic strategies for MM patients include chemotherapy, radiotherapy and targeted therapy [2–4]. However, MM is still incurable by current treatment methods. Uncovering the molecular mechanism behind the progression of MM and intercellular interaction is important to find more effective treatment methods for MM patients.

Non-coding RNAs (ncRNAs) are a class of RNAs that are unable to code proteins generally, and they are abundant in human genome to regulate cellular processes including proliferation, metastasis and apoptosis [5]. Circular RNAs (circRNAs) are a kind of ncRNAs that characterized by covalently closed loop structure [6]. CircRNAs are more stable than linear RNAs and they are resistant to exonuclease due to their loop structure [7]. CircRNAs engaged in the pathogenesis of cancers through serving as microRNAs (miRNAs) sponges to modulate the abundance of downstream genes linked to proliferation, metastasis and apoptosis [8, 9]. The roles of circRNAs in hematological cancers have been reported before [10, 11]. For instance, circ-CBFB contributed to the proliferation ability while suppressed the apoptosis of chronic lymphocytic leukemia cells through targeting miR-607/FZD3/Wnt/beta-catenin signaling [10]. However, the functions of circRNAs in MM remain to be uncovered.

MiRNAs belong to another class of ncRNAs that involved in the progression of cancers through inducing degradation or translational repression of target messenger RNAs (mRNAs) [12]. The dysregulation of miRNAs was involved in the pathogenesis of MM [13, 14]. We concentrated on the role of miR-338-3p. miR-338-3p suppressed the development of many cancers [15–18]. As for MM, Cao et al. reported that miR-338-3p suppressed the proliferation and accelerated the apoptosis of MM cells via CDK4 [19]. Nevertheless, the function of miR-338-3p in MM is largely unexplored.

Bromodomain containing 4 (BRD4) is a crucial epigenetic protein, and it has been reported to elevate the levels of oncogenic proteins and accelerate the progression of cancers [20]. Zheng et al. claimed that H19 accelerated the development of MM through up-regulating BRD4 via sponging miR-152-3p [21]. Here, the direct interaction between miR-338-3p and BRD4 was first found in MM, and the function of BRD4 in MM was investigated.

In this study, circ_0007841 was found to be abnormally up-regulated in MM. Loss-of-function experiments revealed that circ_0007841 silencing blocked the proliferation, cell cycle progression, migration and invasion while induced the apoptosis of MM cells. The underlying mechanisms behind the oncogenic role of circ_0007841 in MM were further explored.

## Materials and methods

### Patients

Plasma cells from MM patients (n = 41) and healthy volunteers (n = 41) in The Fifth Affiliated Hospital of Zhengzhou University were collected to detect the expression of circ_0007841, miR-338-3p and BRD4 via qRT-PCR and Western blot assay.

### Cell culture

MM cell lines (H929 and OPM2) and normal plasma cell line nPCs were purchased from BeNa Culture Collection (Beijing, China) and maintained in Roswell Park Memorial Institute-1640 (RPMI-1640) medium (Gibco, Carlsbad, CA, USA) added with 10% fetal bovine serum (FBS; Gibco), 100 units/mL penicillin and 100 μg/mL streptomycin. Cell culture plates were placed in a 5% CO_2_ incubator at 37 °C, and cells were collected in the log phase of growth.

### Quantitative real-time polymerase chain reaction (qRT-PCR)

After measuring the concentration using NanoDrop 2000 (Invitrogen, Carlsbad, CA, USA), RNA sample (2 ng) was used to synthesize complementary DNA (cDNA) with ReverTra Ace qPCR RT Kit (for circ_0007841, BRD4, glyceraldehyde-3-phosphate dehydrogenase (GAPDH) and U6; Takara, Dalian, China) and All-in-One™ miRNA First stand cDNA Synthesis Kit (for miR-338-3p; GeneCopoeia, Rockville, MD, USA). U6 served as the internal control for miR-338-3p, while GAPDH acted as the internal reference for circ_0007841 and BRD4. PCR amplification reaction was conducted with SYBR Green PCR Master Mix (Applied Biosystems, Foster City, CA, USA) on an ABI 7900 thermocycler (Applied Biosystems). The quantification of circ_0007841, miR-338-3p and BRD4 was carried out with the 2^−ΔΔCt^ method. The specific primers in this study were synthesized from Sangon Biotech (Shanghai, China) and listed as below: circ_0007841 (Forward, 5′-CTAACATCTGTGAAACCATCGT-3′; Reverse, 5′-TCATCACATACACGATAGACTGG-3′), miR-338-3p (Forward, 5′-UCCAGCAUCAGUGAUUUUGUUG-3′; Reverse, 5′-CAACAAAAUCACUGAUGCUGGA-3′), BRD4 (Forward, 5′-GTGGTGCACATCATCCAGTC-3′; Reverse, 5′-CCGACTCTGAGGACGAGAAG-3′), U6 (Forward, 5′-CTCGCTTCGGCAGCACA-3′; Reverse, 5′-AACGCTTCACGAATTTGCGT-3′), GAPDH (Forward, 5′-GCGACACCCACTCCTCCAC-3′; Reverse, 5′-TCCACCACCCTGTTGCTGTAG-3′).

### Cell transfection

Three small interfering RNAs (siRNAs) targeting circ_0007841, including si-circ_0007841#1 (5′-UGUUAGUUGCAAUGAAGAGAG-3′), si-circ_0007841#2 (5′-UAAUGAUCAUGCCAAAUACUC-3′) and si-circ_0007841#3 (5′-UCACAUACACGAUAGACUGGC-3′), its negative control (si-NC), circ_0007841 overexpression plasmid (circ_0007841), its control (Vector), BRD4 overexpression plasmid (BRD4), its control (pcDNA), miR-338-3p mimics (miR-338-3p), its control miR-NC, miR-338-3p inhibitor (in-miR-338-3p) and its control in-miR-NC were obtained from Genepharma (Shanghai, China). MM cells were seeded into 24-well plates at a density of 2 × 10^5^ cells/well overnight, and transfection was conducted with Lipofectamine 3000 (Invitrogen).

### Cell counting kit-8 (CCK8) assay

MM cells were plated in 96-well plates at the density of 5 × 10^3^ cells/well and cultured overnight. After transfection for indicated time points (0 h, 24 h, 48 h or 72 h), MM cells were incubated with 10 μL CCK-8 (Sigma, St. Louis, MO, USA) for 4 h. The absorbance at 450 nm was detected by a microplate reader (BioTek, Winooski, VT, USA).

### Colony formation assay

A total of 150 cells were seeded onto the 6-well plates to settle down. The culture medium was replenished every 4 d. After 2-week incubation, the colonies were immobilized using 4% poly methanol (Sangon Biotech) for 15 min followed by staining using crystal violet (Sangon Biotech).

### Flow cytometry for cell cycle and apoptosis detection

For cell cycle analysis, MM cells were collected using cold phosphate buffer saline (PBS) and then immobilized using 70% cold ethanol solution overnight. Prior to propidium iodide (PI; Solarbio, Beijing, China) staining, RNase was used to remove RNA in the samples. The percentage of MM cells in different phases of cell cycle was detected on the FACSCalibur (Becton–Dickinson, San Jose, CA, USA) and analyzed using Cell Quest software (Becton–Dickinson).

For apoptosis analysis, after transfection for 48 h, MM cells were collected with PBS, and then these cells were suspended in binding buffer. Annexin V-combined fluorescein isothiocyanate (Annexin V-FITC; Solarbio) and PI (Solarbio) were added to the reaction mixture, and MM cells were simultaneously incubated with Annexin V-FITC and PI at 37 °C for 15 min in a dark room. The apoptotic MM cells were identified by FACSCalibur (Becton–Dickinson) and analyzed using Cell Quest software (Becton–Dickinson).

### Transwell assays

In transwell migration assay, cell suspension (MM cells suspended in 100 μL serum-free medium) was added into the upper chambers (Costar, Corning, NY, USA). A total of 600 μL culture medium with 10% FBS was added into the lower chambers. FBS acted as the chemotactic factor in this study. After 24-h incubation, MM cells remained in the upper surface were removed with the cotton swab, and the migrated MM cells were fixed with 4% paraformaldehyde (Sigma) for 20 min and stained with 0.5% crystal violet (Sigma). The number of migrated MM cells in five random visual fields was counted by the microscope (Olympus, Tokyo, Japan).

In transwell invasion assay, the upper chambers were pre-coated with 50 μL Matrigel (Sigma) to mimic the extracellular matrix. The detection of cell invasion was conducted through using these pre-coated transwell chambers following the similar procedure.

### Bioinformatic prediction and dual-luciferase reporter assay

The targets of circ_0007841 and miR-338-3p were predicted by circinteractome and targetscan software, respectively.

The wild-type partial sequence in circ_0007841 that predicted to bind to miR-338-3p, along with the mutant-type sequence with miR-338-3p in circ_0007841 that was synthesized through using Site-directed gene mutagenesis kit (Takara, Dalian, China), was amplified and cloned into pGL3 luciferase reporter vector (Promega, Madison, WI, USA), termed as circ_0007841 WT or circ_0007841 MUT. MM cells were co-transfected with 10 nM miR-NC or miR-338-3p and 40 ng circ_0007841 WT or circ_0007841 MUT. After 48-h transfection, MM cells were harvested and the luciferase activity was detected with the dual-luciferase reporter assay system kit (Promega) using the luminometer (Plate Chameleon V, Hidex, Finland) according to the manufacturer’s instructions. Firefly luciferase activity in each group was normalized to Renilla fluorescence intensity.

The wild-type fragment of BRD4 3′ untranslated region (3′UTR) that predicted to bind to miR-338-3p and the mutant type fragment of BRD4 3′UTR were also amplified and inserted into pGL3 luciferase reporter vector (Promega) to generate BRD4 3′UTR WT and BRD4 3′UTR MUT. Co-transfection of MM cells with BRD4 3′UTR WT or BRD4 3′UTR MUT and miR-NC or miR-338-3p was conducted following the similar procedure.

### RNA-pull down assay

RNA-pull down assay was conducted to test the interaction between circ_0007841 and miR-338-3p. Biotin RNA Labeling Mix (Roche, Shanghai, China) was used in this study. The wild-type and mutant-type binding sites in circ_0007841 that were predicted to bind to miR-338-3p were biotinylated to obtain Bio-circ_0007841 WT and Bio-circ_0007841 MUT. MM cells were disrupted and incubated with Bio-NC, Bio-circ_0007841 WT or Bio-circ_0007841 MUT. The abundance of miR-338-3p was measured by qRT-PCR.

### Western blot assay

Proteins were obtained using whole cell lysis buffer (Roche, Basel, Switzerland) for 30 min on the ice. Protein samples were quantified using Pierce BCA Protein Assay kit (Thermo Fisher Scientific, Rockford, IL, USA). Then, 30 µg of proteins were run on sodium dodecyl sulfate polyacrylamide gel electrophoresis (SDS-PAGE) gel and transferred to the polyvinylidene fluoride (PVDF) membrane (Millipore, Billerica, MA, USA). After blocking with 5% w/v nonfat dry milk for 1 h, primary antibodies were used to probe the indicated proteins followed by incubation with the secondary antibody (ab205718; Abcam, Cambridge, MA, USA). The protein bands were measured using the enhanced chemiluminescent (ECL) system (Beyotime, Shanghai, China) according to the manufacturer’s instructions. Gray analysis was conducted to quantify the expression of proteins using ImageJ software. Primary antibodies, including anti-BRD4 (ab128874), anti-phosphorylated-phosphatidylinositol 3-kinase (anti-p-PI3K; ab70912), anti-PI3K (ab32089), anti-p-AKT serine/threonine kinase (p-AKT; ab38449), anti-AKT (ab64148), anti-CD63 (ab59479), anti-CD81 (ab79559) and anti-β-actin (ab8226), were purchased from Abcam.

### Exosome isolation

Exosome isolation kit (Qiagen, Frankfurt, Germany) was used to extract exosomes from the culture medium of MM cells according to previous studies [22, 23].

### Statistical analysis

All statistical data in three independent experiments were shown as mean ± standard deviation (SD). Data were analyzed using GraphPad Prism 7.0. The differences between two groups or among more than two groups were assessed through using Student’s *t* test or one-way analysis of variance (ANOVA) followed by Tukey’s test. The comparison between groups was considered significant when *P* value less than 0.05. Linear correlation was analyzed using Spearman’s correlation coefficient.

## Results

### Circ_0007841 elevates the malignant behaviors of MM cells

**Fig. 1:**
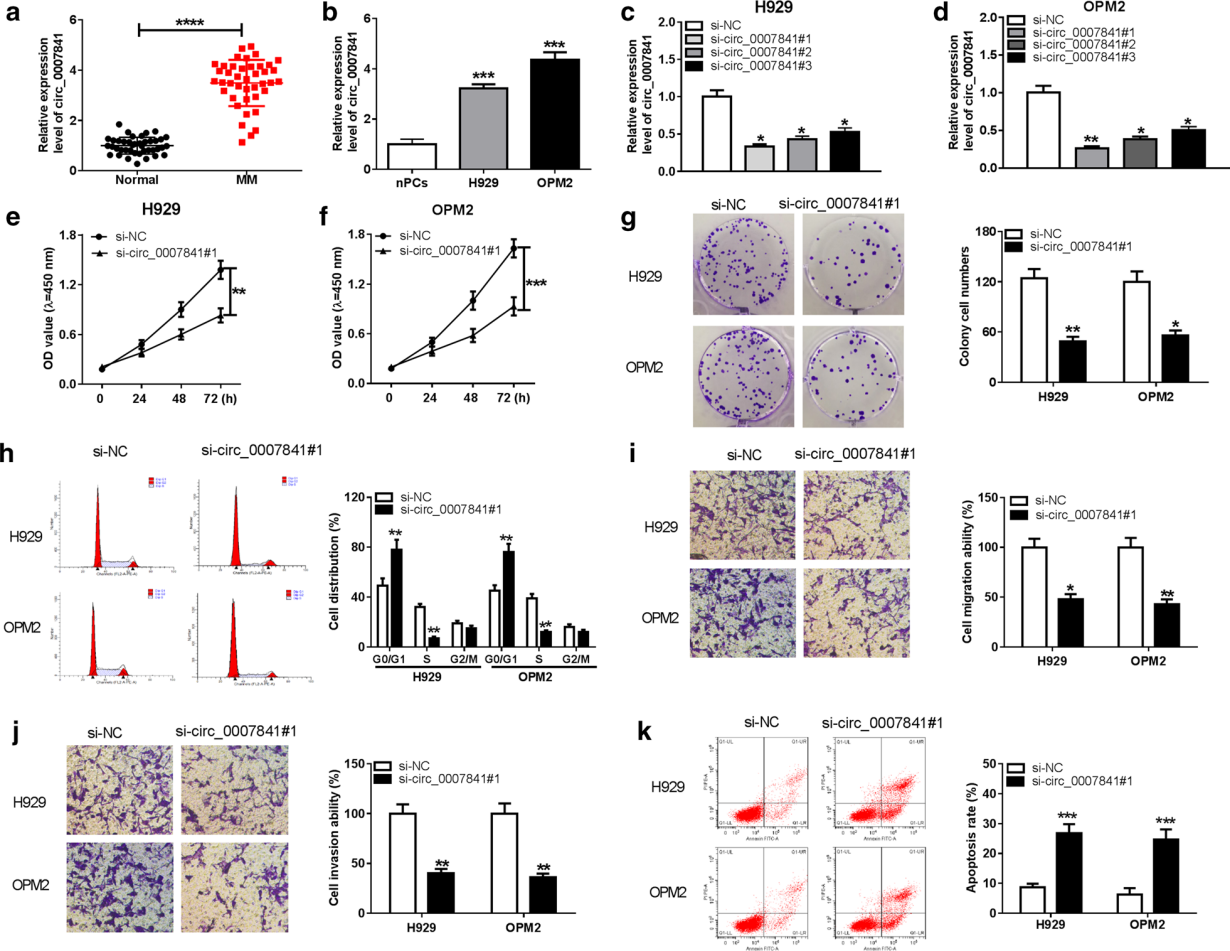
Circ_0007841 elevates the malignant behaviors of MM cells. **a** The enrichment of circ_0007841 was examined in BM-derived plasma cells of MM patients and normal volunteers by qRT-PCR. **b** The expression of circ_0007841 was measured in MM cell lines and normal plasma cell line nPCs by qRT-PCR. **c**, **d** The level of circ_0007841 was detected in H929 and OPM2 cells transfected with si-NC, si-circ_0007841#1, si-circ_0007841#2 or si-circ_0007841#3 by qRT-PCR. **e**–**k** MM cells were transfected with si-NC or si-circ_0007841#1. **e**, **f** CCK8 assay was employed to assess the proliferation ability of MM cells. **g** Colony formation assay was performed for the determination of cell proliferation ability in transfected MM cells. **h** Flow cytometry was carried out to detect the influence of circ_0007841 silencing on the cycle of MM cells. **i**, **j** The metastasis ability of MM cells was evaluated by transwell assays. **k** The apoptosis of MM cells was analyzed by flow cytometry. *P < 0.05, **P < 0.01, ***P < 0.001, ****P < 0.0001

Circ_0007841 was abnormally up-regulated in bone marrow (BM)-derived plasma cells from MM patients compared with that in healthy individuals (Fig. 1a). Meanwhile, the level of circ_0007841 was higher in MM cell lines than that in normal plasma cell line (nPCs, Fig. 1b). The dysregulation of circ_0007841 in MM attached our attention. Circ_0007841 specific small interfering RNAs were used to knockdown circ_0007841 to uncover its biological functions in MM cells. As mentioned in Fig. 1c and d, the level of circ_0007841 was down-regulated with the transfection of si-circ_0007841#1, si-circ_0007841#2 or si-circ_0007841#3. Among these three siRNAs, si-circ_0007841#1 was chose for the following assays due to its highest knockdown efficiency (Fig. 1c, d). Cell proliferation was assessed through CCK8 assay, colony formation assay and flow cytometry. According to the results of CCK8 assay, si-circ_0007841#1 transfection significantly inhibited the proliferation of MM cells (Fig. 1e, f). The number of colonies was markedly reduced with the knockdown of circ_0007841 compared with si-NC group (Fig. 1g). The cell cycle of MM cells was arrested in G1/S transition in si-circ_0007841#1 group than that in si-NC group (Fig. 1h). These findings together demonstrated that circ_0007841 silencing hampered the proliferation ability in MM cells. What’s more, circ_0007841 interference notably suppressed the migration and invasion of MM cells via transwell migration and invasion assays (Fig. 1i, j). The apoptosis rate of MM cells was increased in si-circ_0007841#1 group compared with that in si-NC group (Fig. 1k). Overall, circ_0007841 accelerated the proliferation, cell cycle progression and metastasis and inhibited the apoptosis of MM cells.

### miR-338-3p could directly interact with circ_0007841 in MM cells

**Fig. 2:**
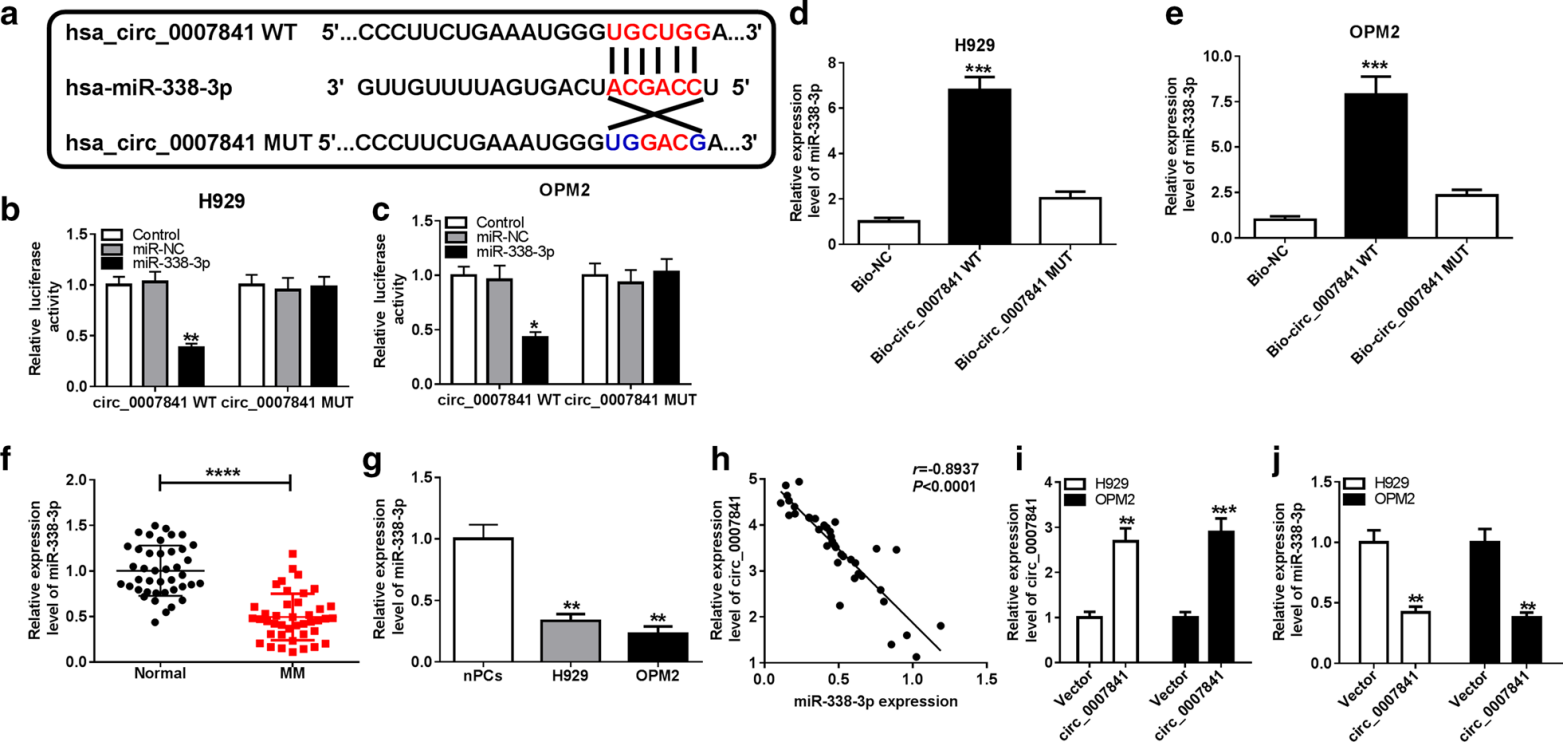
miR-338-3p could directly interact with circ_0007841 in MM cells. **a** miR-338-3p was predicted as a candidate target of circ_0007841 by circinteractome software. **b**, **c** Dual-luciferase reporter assay was conducted to verify whether miR-338-3p could bind to circ_0007841 in MM cells. **d**, **e** RNA-pull down assay was performed to confirm the target relationship between miR-338-3p and circ_0007841 in MM cells. **f**, **g** The expression of miR-338-3p was detected in BM-derived plasma cells of MM patients and healthy volunteers, MM cells and nPCs cells by qRT-PCR. **h** The correlation between the expression of miR-338-3p and circ_0007841 was analyzed using Spearman’s coefficient. **i**, **j** The abundance of circ_0007841 and miR-338-3p was examined in H929 and OPM2 cells transfected with Vector or circ_0007841 by qRT-PCR. *P < 0.05, **P < 0.01, ***P < 0.001, ****P < 0.0001

To address the mechanism by which circ_0007841 functioned in MM cells, circinteractome website was used to seek the targets of circ_0007841. As shown in Fig. 2a, miR-338-3p possessed the complementary sites with circ_0007841. The luciferase activity was dramatically reduced in circ_0007841 WT group when co-transfected with miR-338-3p, suggesting the target relationship between circ_0007841 and miR-338-3p in MM cells (Fig. 2b, c). We also constructed mutant type luciferase plasmid (circ_0007841 MUT) to investigate if “UGCUGG” in circ_0007841 was the binding sequence with miR-338-3p. The luciferase intensity remained unaffected in circ_0007841 MUT group with the co-transfection of miR-NC or miR-338-3p (Fig. 2b, c), suggested that circ_0007841 bound to miR-338-3p via its “UGCUGG” sequence. RNA-pull down assay revealed that miR-338-3p could be pulled-down when using Bio-circ_0007841 WT, proving the target relationship between miR-338-3p and circ_0007841 (Fig. 2d, e). An obvious decrease in the level of miR-338-3p was observed in BM-derived plasma cells from MM patients in contrast to that in normal volunteers (Fig. 2f). Additionally, there was a prominent reduction in the expression of miR-338-3p in MM cell lines than that in nPCs cell line (Fig. 2g). The expression of miR-338-3p was negatively correlated with the level of circ_0007841 in BM-derived plasma cells from MM patients (Fig. 2h). The overexpression efficiency of circ_0007841 was high in MM cells, and circ_0007841 accumulation caused a notable decrease in the level of miR-338-3p in MM cells (Fig. 2i, j). In summary, circ_0007841 could inversely regulate the expression of miR-338-3p through direct interaction.

### Circ_0007841 plays an oncogenic role through targeting miR-338-3p in MM cells

**Fig. 3:**
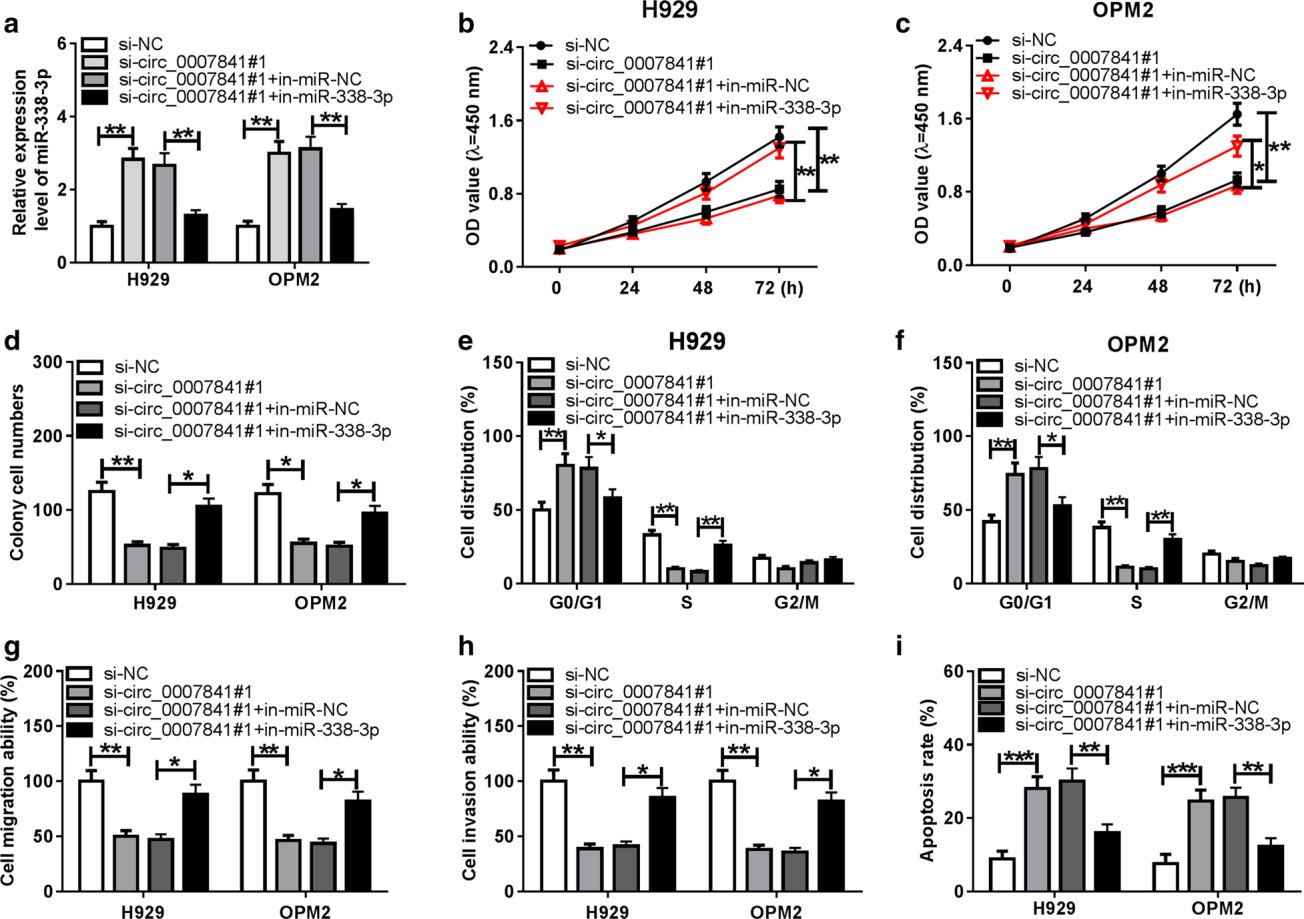
Circ_0007841 plays an oncogenic role through targeting miR-338-3p in MM cells. **a**–**i** MM cells were transfected with si-NC, si-circ_0007841#1, si-circ_0007841#1 + in-miR-NC or si-circ_0007841#1 + in-miR-338-3p. **a** The level of miR-338-3p was examined in MM cells by qRT-PCR assay. **b**, **c** The proliferation of MM cells was measured through conducting CCK8 assay. **d** The proliferation capacity in transfected MM cells was assessed by colony formation assay. **e**, **f** The percentage of MM cells in G0/G1, S or G2/M phase was analyzed using flow cytometry. **g**, **h** The migration and invasion abilities of MM cells were evaluated by transwell assays. **i** The apoptosis rate of MM cells in different groups was analyzed by flow cytometry. *P < 0.05, **P < 0.01, ***P < 0.001

To disclose if circ_0007841 exerted its oncogenic role through targeting miR-338-3p, we conducted rescue experiments through co-transfecting H929 and OPM2 cells with si-NC, si-circ_0007841#1, si-circ_0007841#1 + in-miR-NC or si-circ_0007841#1 + in-miR-338-3p. As mentioned in Fig. 3a, si-circ_0007841#1 transfection increased the level of miR-338-3p, and the introduction of in-miR-338-3p reversed the influence of circ_0007841 silencing in the expression of miR-338-3p. Si-circ_0007841#1-mediated inhibitory effect on the proliferation of MM cells was counteracted by the interference of miR-338-3p via CCK8 assay (Fig. 3b, c). Circ_0007841 silencing restrained the colony formation ability, while the addition of miR-338-3p inhibitor partly recovered the colony formation ability in MM cells (Fig. 3d). Additionally, cell cycle of MM cells was arrested at G1/S transition in si-circ_0007841#1 group, and this suppressive impact in the cell cycle of MM cells was attenuated by the addition of in-miR-338-3p (Fig. 3e, f). The migration and invasion of MM cells were suppressed by the knockdown of circ_0007841, and the metastasis ability was recovered in si-circ_0007841#1 and in-miR-338-3p co-transfected group (Fig. 3g, h). Si-circ_0007841#1-induced apoptosis of MM cells was attenuated by the addition of in-miR-338-3p (Fig. 3i). Overall, circ_0007841 could promote the malignant potential of MM cells through sponging miR-338-3p.

### BRD4 is validated as a target of miR-338-3p in MM cells

**Fig. 4:**
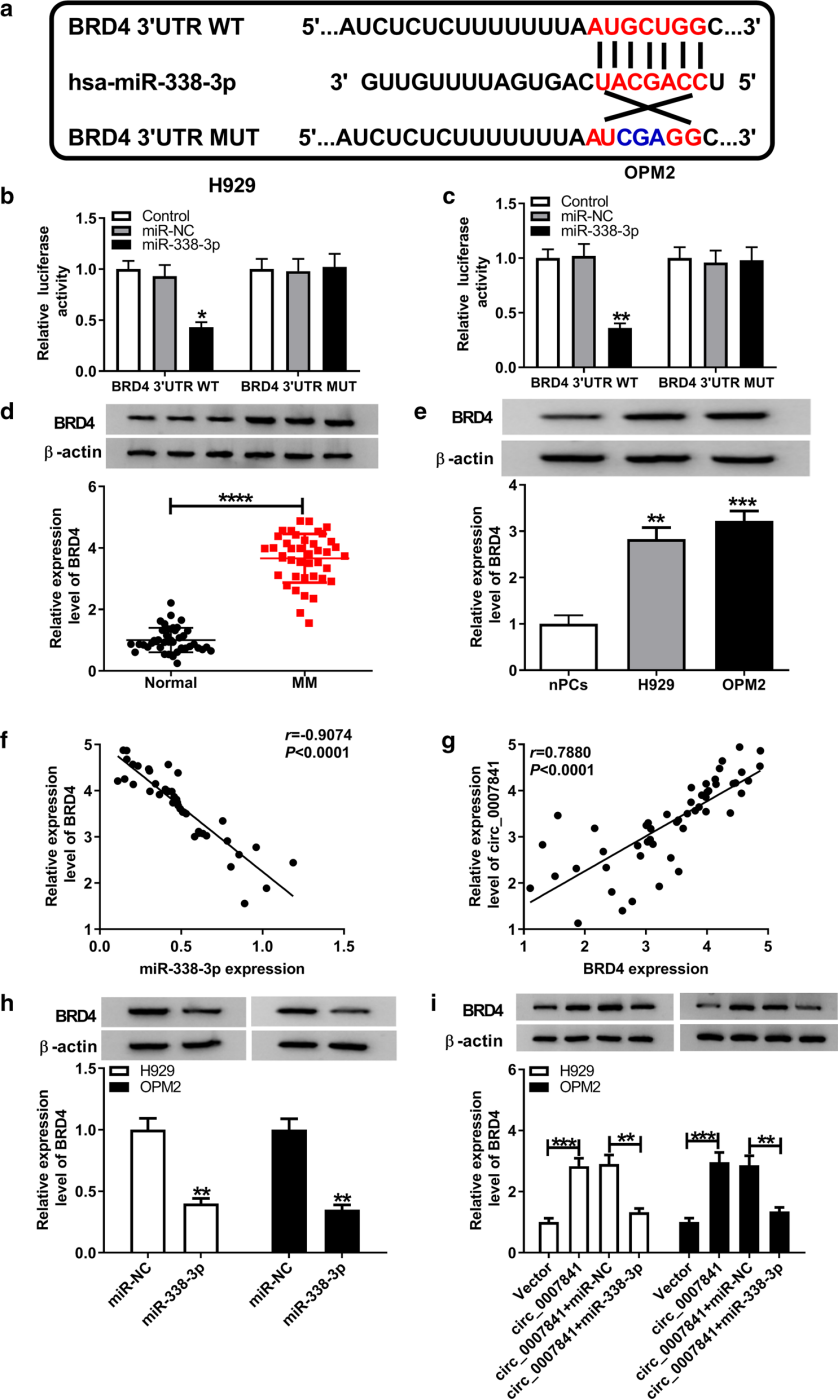
BRD4 is validated as a target of miR-338-3p in MM cells. **a** The complementary sites between miR-338-3p and the 3′UTR of BRD4 were predicted by targetscan software. **b**, **c** The luciferase activity was measured in H929 and OPM2 cells transfected with miR-NC or miR-338-3p and BRD4 3′UTR WT or BRD4 3′UTR MUT. **d** The protein level of BRD4 in BM-derived plasma cells of MM patients and healthy volunteers was detected by Western blot assay. **e** The level of BRD4 in H929, OPM2 and nPCs cells was evaluated by Western blot assay. **f**, **g** The linear relationship between BRD4 and miR-338-3p or circ_0007841 was analyzed using Spearman’s coefficient. **h** The expression of BRD4 was detected in MM cells transfected with miR-NC or miR-338-3p by Western blot assay. **i** The protein level of BRD4 was detected in MM cells transfected with Vector, circ_0007841, circ_0007841 + miR-NC or circ_0007841 + miR-338-3p by Western blot assay. *P < 0.05, **P < 0.01, ***P < 0.001, ****P < 0.0001

BRD4 was predicted as a direct target of miR-338-3p by targetscan database, and the wild type or the mutant type binding sequence between miR-338-3p and BRD4 was shown in Fig. 4a. As exhibited in Fig. 4b, c, the luciferase activity was markedly decreased in miR-338-3p and BRD4 3′untranslated region (3′UTR) WT co-transfected group, while miR-338-3p transfection had no effect on the luciferase activity in BRD4 3′UTR MUT group compared with that in miR-NC and BRD4 3′UTR MUT co-transfected group, suggesting the interaction between BRD4 and miR-338-3p. BRD4 was conspicuously up-regulated in BM-derived plasma cells of MM patients compared with that in healthy individuals (Fig. 4d). Meanwhile, BRD4 was also found to be up-regulated in MM cell lines than that in nPCs cells (Fig. 4e). The expression correlation between BRD4 and circ_0007841 or miR-338-3p was analyzed using Spearman’s correlation coefficient. As shown in Fig. 4f, g, there was an inverse correlation between the levels of BRD4 and miR-338-3p, while the expression of BRD4 was positively correlated with the level of circ_0007841. miR-338-3p overexpression significantly down-regulated the expression of BRD4 in MM cells, suggesting the negative regulatory relationship between BRD4 and miR-338-3p in MM cells (Fig. 4h). Circ_0007841 and miR-338-3p were co-transfected into MM cells to uncover the relationship among circ_0007841, miR-338-3p and BRD4. As presented in Fig. 4i, circ_0007841 overexpression up-regulated the level of BRD4, and the expression of BRD4 was decreased in circ_0007841 and miR-338-3p co-transfected group. Collectively, BRD4 was a target of miR-338-3p, and circ_0007841 could elevate the expression of BRD4 through sponging miR-338-3p.

### BRD4 overexpression attenuates the effects of miR-338-3p accumulation on MM cells

**Fig. 5:**
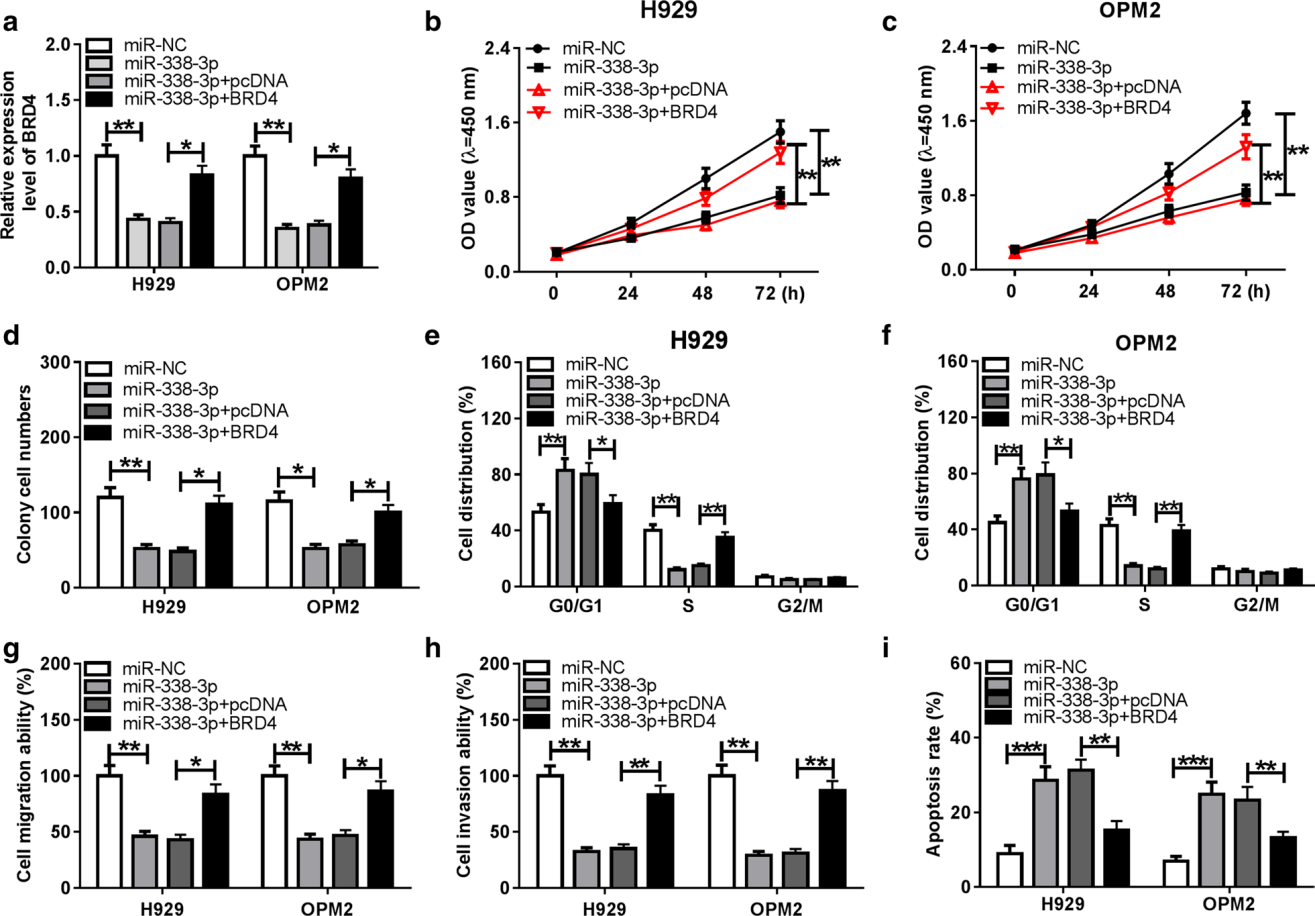
BRD4 overexpression attenuates the effects of miR-338-3p accumulation on MM cells. **a**–**i** MM cells were transfected with miR-NC, miR-338-3p, miR-338-3p + pcDNA or miR-338-3p + BRD4. **a** qRT-PCR was employed to measure the expression of BRD4 in MM cells. **b**, **c** CCK8 assay was applied to assess the proliferation ability of MM cells. **d** Colony formation assay was performed to analyze the influences of miR-338-3p and BRD4 on the proliferation of MM cells. **e**, **f** Flow cytometry was conducted to detect the cell cycle of MM cells. **g**, **h** Transwell assays were performed to detect the metastasis of MM cells. **i** The apoptosis rate of MM cells was examined by flow cytometry. *P < 0.05, **P < 0.01, ***P < 0.001

miR-338-3p and BRD4 were co-transfected into MM cells to explore whether miR-338-3p exerted an anti-tumor role in MM cells through targeting BRD4. As shown in Fig. 5a, the addition of BRD4 overexpression plasmid recovered the expression of BRD4 in MM cells that was down-regulated by the accumulation of miR-338-3p. miR-338-3p overexpression inhibited the proliferation, cell cycle and metastasis of MM cells, and these inhibitory effects were attenuated by the addition of BRD4 overexpression plasmid (Fig. 5b–h). The apoptosis of MM cells was induced by the transfection of miR-338-3p, and the introduction of BRD4 overexpression plasmid recovered the viability of MM cells (Fig. 5i). In conclusion, miR-338-3p accumulation restrained the malignant behaviors of MM cells through targeting BRD4.

### Circ_0007841 activates PI3K/AKT signal pathway through targeting miR-338-3p/BRD4 axis

**Fig. 6:**
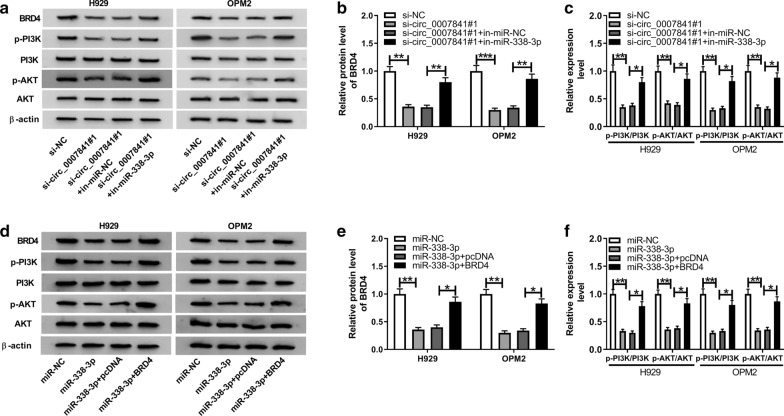
Circ_0007841 activates PI3K/AKT signal pathway through targeting miR-338-3p/BRD4 axis. **a**–**c** Western blot assay was performed to detect the levels of BRD4 and PI3K/AKT signaling-related proteins in MM cells transfected with si-NC, si-circ_0007841#1, si-circ_0007841#1 + in-miR-NC or si-circ_0007841#1 + in-miR-338-3p, and gray analysis was used to assess the abundance of these proteins. **d**–**f** The expression of BRD4 and PI3K/AKT signaling-associated proteins in MM cells transfected with miR-NC, miR-338-3p, miR-338-3p + pcDNA or miR-338-3p + BRD4 was examined by Western blot assay. *P < 0.05, **P < 0.01, ***P < 0.001

The activation of PI3K/AKT signal pathway is linked to the promotion of cell proliferation and metastasis and the inhibition of cell apoptosis. Herein, we examined the phosphorylation levels of PI3K and AKT to illustrate the influence of circ_0007841/miR-338-3p/BRD4 axis in the activation of PI3K/AKT signaling. Circ_0007841 silencing down-regulated the level of BRD4, and the level of BRD4 was recovered in si-circ_0007841#1 and in-miR-338-3p co-transfected group (Fig. 6a, b). The activation of PI3K/AKT signaling was suppressed with the silencing of circ_0007841, and the addition of in-miR-338-3p recovered the phosphorylation levels of PI3K and AKT (Fig. 6a, c). Meanwhile, H929 and OPM2 cells were transfected with miR-NC, miR-338-3p, miR-338-3p + pcDNA or miR-338-3p + BRD4. As mentioned in Fig. 6d, e, miR-338-3p overexpression down-regulated the level of BRD4, and the introduction of BRD4 overexpression plasmid regained the level of BRD4 in MM cells. The addition of BRD4 alleviated the inhibitory influence of miR-338-3p overexpression on the activation of PI3K/AKT signaling in MM cells (Fig. 6d, f). Taken together, circ_0007841 accelerated the progression of MM through miR-338-3p/BRD4/PI3K/AKT axis.

### Mesenchymal stromal cells (MSCs)-generated exosomes accelerate the malignant potential of MM cells via circ_0007841

**Fig. 7:**
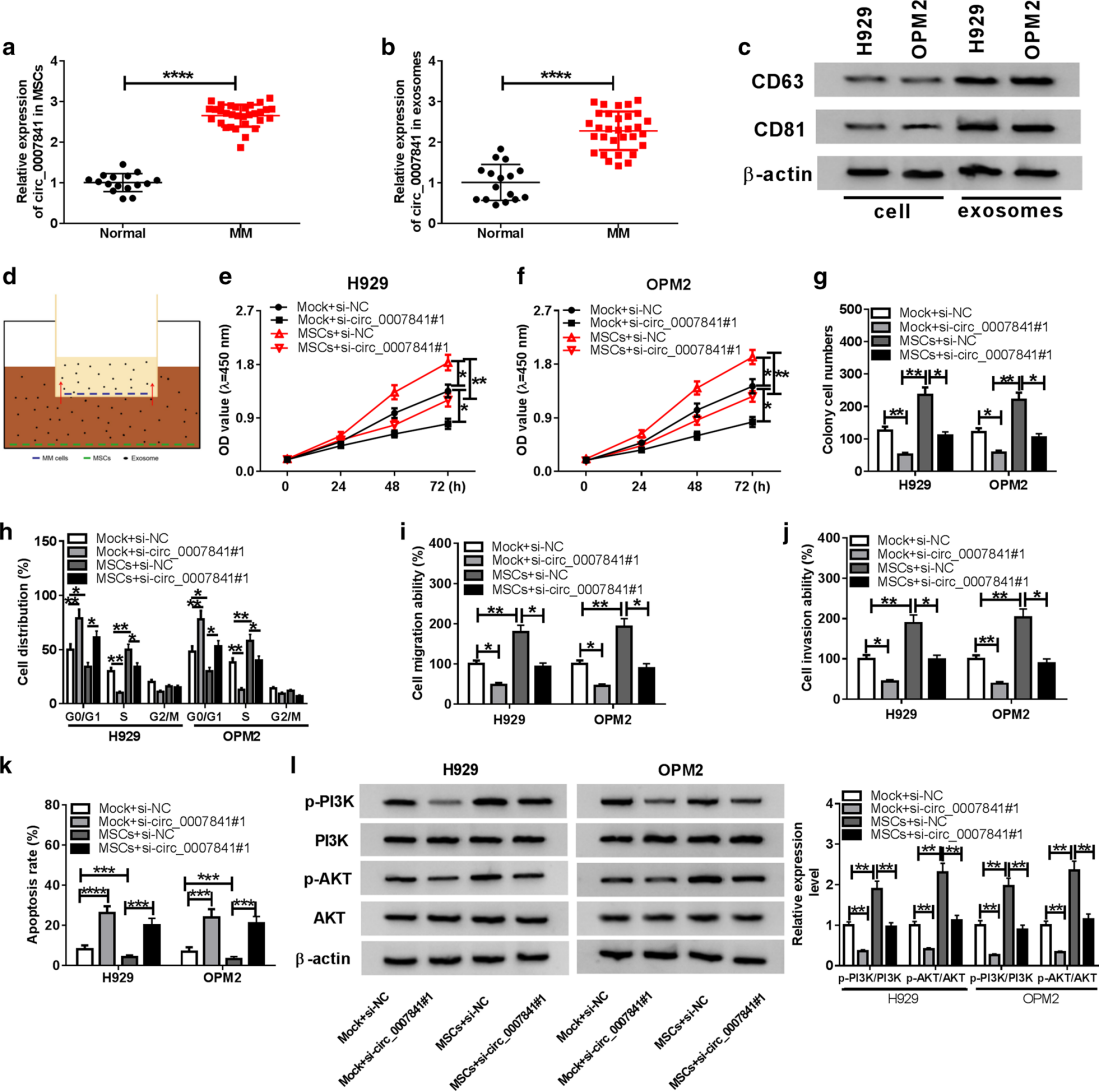
MSCs-generated exosomes accelerate the malignant potential of MM cells via circ_0007841. **a**, **b** The expression of circ_0007841 was detected in the MSCs and MSCs-generated exosomes from the adjacent tissues of MM and normal tissues by qRT-PCR. **c** Western blot assay was performed to detect the protein levels of exosome-related markers, including CD63 and CD81, in cell lysate and exosomes. **d** The model showed that MM cells were co-cultured with MSCs, and only exosomes could move from the lower chambers to the upper chambers. **e**–**l** MM cells transfected with si-NC or si-circ_0007841#1 were co-cultured with MSCs or not. **e**, **f** CCK8 assay was performed to assess the proliferation of MM cells. **g** The proliferation of MM cells was evaluated by colony formation assay. **h** The cell cycle of MM cells was detected through conducting flow cytometry. **i**, **j** The abilities of migration and invasion of MM cells were assessed by transwell assays. **k** The apoptosis of MM cells was examined through performing flow cytometry. **l** The levels of p-PI3K, PI3K, p-AKT and AKT were detected in MM cells by Western blot assay. *P < 0.05, **P < 0.01, ***P < 0.001, ****P < 0.0001

MSCs exert crucial roles in the progression of MM. Herein, we explored whether exosomes derived from MSCs could regulate the proliferation, cell cycle, metastasis and apoptosis of MM cells via circ_0007841. MSCs were isolated from the adjacent tissues of MM and normal tissues. The expression of circ_0007841 was higher in MSCs and MSCs-derived exosomes from adjacent tissues than that in normal tissues (Fig. 7a, b). The markers of exosomes (CD63 and CD81) were notably up-regulated in exosomes of MSCs instead of cell lysate (Fig. 7c). As mentioned in Fig. 7d, we established a working model as previously described to explore if MSCs-derived exosomes could regulate the proliferation, cell cycle, motility and apoptosis of MM cells [24]. In this model, only exosomes could be transmitted through the filter to the upper chambers. As presented in Fig. 7e–k, si-circ_0007841#1 transfection inhibited the malignant behaviors of MM cells in Mock + si-circ_0007841#1 group compared with that in Mock + si-NC group. Besides, MSCs-derived exosomes (MSCs + si-NC group) promoted the proliferation, cell cycle, metastasis and inhibited the apoptosis of MM cells than that in Mock + si-NC group, and these effects were attenuated by the silencing the circ_0007841, suggested that MSCs-derived exosomes could promote the progression of MM via circ_0007841. What’s more, the exosomes generated from MSCs accelerated the activation of PI3K/AKT signaling, while this effect was counteracted with the transfection of si-circ_0007841#1 (Fig. 7l). Collectively, MSCs-derived exosomes could facilitate the progression of MM via circ_0007841.

## Discussion

MM is an incurable cancer currently. Because many MM patients were diagnosed at late stage, the treatment outcomes of MM patients were unsatisfactory [25]. Therefore, finding crucial markers in MM is urgent to improve the prognosis of MM patients.

CircRNAs are featured by closely loop structure and they are widely distributed in human tissues. Due to the stability and the universality of the distribution, circRNAs are identified as ideal biomarkers for human cancers and other diseases [26]. For example, the high expression of circ_0004277 was associated with the better prognosis of AML patients [27]. Xia et al. claimed that circ-CBFB was highly expressed in chronic lymphocytic leukemia, and circ-CBFB accelerated the proliferation and suppressed the apoptosis of chronic lymphocytic leukemia cells [10]. Circ_0007841 was found to be overexpressed in BM-derived plasma cells of MM patients and MM cells. Further studies suggested that circ_0007841 promoted the proliferation, cell cycle, metastasis and inhibited the apoptosis of MM cells. These findings demonstrated that circ_0007841 might be an important biomarker for MM patients, which was in agreement with the former article [28]. However, the regulatory mechanism by which circ_0007841 promoted the progression of MM was unclear.

MiRNAs are single-stranded ncRNAs, and they are implicated in cell proliferation, metastasis and apoptosis through base pairing with mRNAs [29]. Additionally, circRNAs could act as miRNAs sponges to function [30]. miR-338-3p played a tumor suppressor role in multiple cancers. For instance, Sui et al. proved that miR-338-3p suppressed the development of thyroid cancer via AKT3 [31]. Jin et al. found that miR-338-3p played an anti-tumor role in breast cancer via SOX4 [32]. Xue et al. demonstrated that miR-338-3p suppressed the metastasis of colorectal cancer cells through targeting smoothened [33]. miR-338-3p was also found to be down-regulated in MM, and it suppressed the proliferation and facilitated the apoptosis of MM cells via CDK4 [19]. miR-338-3p was identified as a novel target of circ_0007841 in MM cells in our study. Subsequently, rescue experiments were performed to explore whether circ_0007841 functioned through sponging miR-338-3p. We found that si-circ_0007841#1-mediated effects in MM cells were alleviated by the transfection of in-miR-338-3p, suggested that circ_0007841 acted as an oncogene in MM through targeting and down-regulating miR-338-3p.

To uncover the potential mechanism that was responsible for the functions of miR-338-3p in MM cells, the downstream genes of miR-338-3p were searched using targetscan software. BRD4 was confirmed as a candidate gene of miR-338-3p. BRD4 was regulated by circ_0007841/miR-338-3p axis in MM cells. High expression of BRD4 promoted the progression of high-grade serous ovarian cancer [34]. Besides, BRD4 was found to be a target of H19/miR-152-3p axis to promote the progression of MM [21]. Rescue experiments revealed that BRD4 overexpression attenuated the inhibitory effects of miR-338-3p transfection on the proliferation, cell cycle and metastasis and the promoting effect on the apoptosis of MM cells, proved that BRD4 acted as a target of miR-338-3p to promote the progression of MM.

PI3K/AKT signal pathway was related to cell proliferation, viability, apoptosis and autophagy. Accumulating articles have reported the association between the pro-proliferative influence of circRNAs in cancer cells and the activation of PI3K/AKT pathway. For example, circ-IGF1R promoted the proliferation and blocked the apoptosis of hepatocellular carcinoma cells through activating PI3K/AKT pathway [35]. Liu et al. found that circ_8073 accelerated the proliferation of caprine endometrial epithelial cells through activating PI3K/AKT/mTOR pathway via miR-449a/CEP55 axis [36]. Further studies demonstrated that the activation PI3K/AKT signaling could be regulated by circ_0007841/miR-338-3p/BRD4 axis in MM cells. Additionally, we found that MSCs-generated exosomes could accelerate the progression of MM via circ_0007841.

## Conclusions

In summary, our studies identified a novel circ_0007841/miR-338-3p/BRD4 signal axis in MM. Circ_0007841 facilitated the proliferation, cell cycle progression and metastasis and inhibited the apoptosis of MM cells through acting as a decoy of miR-338-3p to up-regulate BRD4 level. Circ_0007841/miR-338-3p/BRD4 axis might be a promising therapeutic target for MM patients.

## Data Availability

The data sets used and/or analyzed during the current study are available from the corresponding author on reasonable request.

## Abbreviations

MM: multiple myeloma
qRT-PCR: quantitative real-time polymerase chain reaction
BRD4: bromodomain containing 4
CCK8: cell counting kit-8
p-PI3K: phosphorylated-phosphatidylinositol 3-kinase
BM: bone marrow
MSCs: mesenchymal stromal cells
ncRNAs: non-coding RNAs
circRNAs: circular RNAs
miRNAs: microRNAs
mRNAs: messenger RNAs
siRNAs: small interfering RNAs
PVDF: polyvinylidene fluoride
MSCs: mesenchymal stromal cells
